# Selective Histone Deacetylase 6 Inhibition Normalizes B Cell Activation and Germinal Center Formation in a Model of Systemic Lupus Erythematosus

**DOI:** 10.3389/fimmu.2019.02512

**Published:** 2019-10-25

**Authors:** Jingjing Ren, Michelle D. Catalina, Kristin Eden, Xiaofeng Liao, Kaitlin A. Read, Xin Luo, Ryan P. McMillan, Matthew W. Hulver, Matthew Jarpe, Prathyusha Bachali, Amrie C. Grammer, Peter E. Lipsky, Christopher M. Reilly

**Affiliations:** ^1^Department of Biomedical Sciences and Pathobiology, Virginia-Maryland College of Veterinary Medicine, Virginia Polytechnic Institute and State University, Blacksburg, VA, United States; ^2^AMPEL BioSolutions, Charlottesville, VA, United States; ^3^RILITE Research Institute, Charlottesville, VA, United States; ^4^Virginia Tech Carilion Research Institute, Virginia Polytechnic Institute and State University, Blacksburg, VA, United States; ^5^Department of Human Nutrition, Foods, and Exercise, Virginia Polytechnic Institute and State University, Blacksburg, VA, United States; ^6^Regenacy Pharmaceuticals, Waltham, MA, United States; ^7^Edward Via College of Osteopathic Medicine, Blacksburg, VA, United States

**Keywords:** lupus nephritis (LN), systemic lupus erythematosus (SLE), histone deacetylase (HDAC) 6, RNA-seq, B cell, germinal center (GC), B cell signaling

## Abstract

Autoantibody production by plasma cells (PCs) plays a pivotal role in the pathogenesis of systemic lupus erythematosus (SLE). The molecular pathways by which B cells become pathogenic PC secreting autoantibodies in SLE are incompletely characterized. Histone deactylase 6 (HDAC6) is a unique cytoplasmic HDAC that modifies the interaction of a number of tubulin- associated proteins; inhibition of HDAC6 has been shown to be beneficial in murine models of SLE, but the downstream pathways accounting for the therapeutic benefit have not been clearly delineated. In the current study, we sought to determine whether selective HDAC6 inhibition would abrogate abnormal B cell activation in SLE. We treated NZB/W lupus mice with the selective HDAC6 inhibitor, ACY-738, for 4 weeks beginning at 20 weeks-of age. After only 4 weeks of treatment, manifestation of lupus nephritis (LN) were greatly reduced in these animals. We then used RNAseq to determine the genomic signatures of splenocytes from treated and untreated mice and applied computational cellular and pathway analysis to reveal multiple signaling events associated with B cell activation and differentiation in SLE that were modulated by HDAC6 inhibition. PC development was abrogated and germinal center (GC) formation was greatly reduced. When the HDAC6 inhibitor-treated lupus mouse gene signatures were compared to human lupus patient gene signatures, the results showed numerous immune, and inflammatory pathways increased in active human lupus were significantly decreased in the HDAC6 inhibitor treated animals. Pathway analysis suggested alterations in cellular metabolism might contribute to the normalization of lupus mouse spleen genomic signatures, and this was confirmed by direct measurement of the impact of the HDAC6 inhibitor on metabolic activities of murine spleen cells. Taken together, these studies show HDAC6 inhibition decreases B cell activation signaling pathways and reduces PC differentiation in SLE and suggest that a critical event might be modulation of cellular metabolism.

## Introduction

Systemic lupus erythematosus (SLE) is a multi-organ autoimmune disease characterized by the production of pathogenic antibodies with the formation of immune complexes that can be deposited in various tissues. Plasma cells (PCs) are differentiated B cells responsible for the production of antibodies that provide defense from invading foreign pathogens. After activation, B cells either (a) form short-lived extrafollicular plasmablasts that are critical for early protective immunity or (b) enter specialized regions of secondary lymphoid tissue that facilitate T cell: B cell collaboration, either germinal centers (GCs) or extra-follicular foci and undergo extensive proliferation eventually becoming PC that produce high avidity antibody via somatic hypermutation. In lupus, PCs differentiated from active B cells produce autoantibodies such as anti-dsDNA and anti-RNA–binding proteins, which bind self-antigens forming immune complex that deposit in blood vessels and renal glomeruli leading to vasculitis and nephritis. Although much is known about the mechanisms regulating T cell: B cell collaboration and PC generation in SLE, many details of the intracellular event regulating this process have not yet been delineated.

Post-translational modification (PTM) of proteins is an important means to regulate protein: protein interactions and downstream cellular functions. In SLE, PTM modified self-proteins play important roles in induction and initiation of autoimmune response by creating neo-epitopes (1, 2). The isotype of autoantibodies is determined by the modified histone proteins in murine and human lupus (3). Among the various PTMs of proteins, acetylation plays a major role. Studies also showed the significant enrichment of lysine acetylation proteins in SLE, which widely contributed to a variety of cellular functions (4). Acetylation/deacetylation events are reversible PTM on lysine residues of histone and non-histone proteins and are essential for specific protein: protein interactions and in the nucleus for gene regulation. These reactions are typically catalyzed by enzymes with histone acetyltransferase (HAT) or histone deacetylase (HDAC) activity. HDACs are classified into four subclasses: three Zn^2+^-dependent classes (I, II, and IV), and one NAD+-dependent class III. Class II is subdivided into class IIa and class IIb. HDAC6 belongs to HDAC class IIb and is largely cytoplasmic in location. It is associated with non-histone substrates, including α-tubulin, heat shock protein 90 (HSP90), and cortactin and others and has been shown to modulate immune cell function in various ways, including modifying BCL6 function and B cell maturation (5).

We have previously shown that the selective HDAC6 inhibitor ACY-738 given to pre-disease lupus-prone NZB/W mice prevented the onset of lupus nephritis (LN). In our current studies, we treated NZB/W mice for only 4 weeks after disease onset and sought to determine mechanisms by which this cytoplasmic HDAC inhibitor might alter the cellular functions involved in lupus pathogenesis and especially the maintenance of GC and PC generation. To accomplish this, we assessed changes in the mRNA transcriptome mediated by selective HDAC6 inhibition using RNA-Sequencing (RNA-seq) analysis of whole splenocytes. We found that HDAC6 inhibition in NZB/W mice led to global changes in gene expression. Phenotypically, we found that decreased glomerulonephritis was coupled with reduced IgG and C3 deposition and decreased GC and PC populations. Furthermore, we observed reduced B cell activation following HDAC6 inhibitor treatment. Underlying this was a change in cellular metabolism. Taken together, these data indicate that targeting autoreactive B cells through increased acetylation may limit cell activation and differentiation in lupus, thereby provide therapeutic benefit.

## Materials and Methods

### Mice and ACY-738 Treatment

Female New Zealand Black/White F1 (NZB/WF1/J) (NZB/W) were obtained from The Jackson Laboratory (Bar Harbor, ME, USA). For ACY-738 treatment, NZB/W mice were given a diet mixed with or without 200 mg/kg ACY-738, which was purchased from Envigo (form 8640, Huntingdon, UK). Treatment started at 20 weeks-of-age when the animals began to show signs of mild proteinuria (30 mg/dl by dipstick analysis). All animals were allowed food and water *ad libitum*. Treatment was continued for 4 weeks at which time, animals were euthanized.

### Immunofluorescence

At the termination of the experiment, the spleens and kidneys were removed. One portion of the spleen and the kidney was embedded in Tissue-TekVR optimal cutting temperature compound (O.C.T.TM) (Sakura Finetek, Torrance, CA, USA) and frozen rapidly in a freezing bath of dry ice and 2-methylbutane. Frozen OCT samples were cryosectioned into 5 and 10 μm sections, respectively. Frozen slides were warmed to room temperature and allowed to dry for 30 min, followed by fixation in cold acetone at room temperature for 10 min. After washing in PBS, slides were blocked with PBS containing 1% bovine serum albumin (BSA) and anti-mouse CD16/32 for 20 min at room temperature. Slides were then incubated with a fluorochrome-conjugated antibody mixture for 1 h at room temperature in a dark humid box. Slides were mounted with Prolong Gold containing DAPI (Life Technologies, Carlsbad, CA, USA). The following anti-mouse antibodies were used in immunohistochemical analysis: anti-IgG-phycoerythrin (PE) (eBioscience, Santa Clara, CA, USA) and anti-C3-fluorescein isothiocyanate (FITC) (Cedarlanelabs, Burlington, Canada), anti-IgD-phycoerythrin (PE) (eBioscience, Santa Clara, CA, USA), anti-CD3-APC (Biolegend, San Diego, CA, USA), Peanut Agglutinin (PNA)-fluorescein isothiocyanate (FITC) (Burlingame, CA, USA), anti-CD138- phycoerythrin (PE) (eBioscience, Santa Clara, CA, USA) and anti-IgM-V450 (BD bioscience, Franklin Lakes, NJ). Slides stained with antibodies were read and visualized with an EVOSVR FL microscope (Advanced Microscopy Group, Grand Island, NY, USA) and a x40 and x20 objective for kidney and for spleen, respectively. Six randomly selected glomeruli from each sample were pictured and then analyzed by using ImageJ software (National Institutes of Health, Rockville, MD, USA) to calculate the deposition of IgG and C3. For spleens, a total of 20 spots were imaged for each group of 4 mice, with five random spots imaged from each mouse, from which representative figures were selected.

### mRNA-Isolation and Sequencing

Total RNA was isolated from whole splenocytes using the miRNeasy Mini Kit (Qiagen, Germantown, MD, USA) per manufacturer's instructions. To remove residual amounts of DNA contamination in isolated RNA, on-column DNase digestion with RNase-Free DNase was performed. The RNA concentration was quantified using a NanoDrop 2000. Total RNA was sent to Beckman Coulter (Danvers, MA, USA) for 2 × 100 bp paired-end Illumina RNA sequencing with an average of 40 million reads per sample. Sequencing data (FASTQ files) was trimmed for both adaptor sequences and quality using a combination of ea-utils and Btrim (6, 7). Sequencing reads were then aligned to the genome (Ensembl.org 38.74) using Bowtie2/Tophat2 (8, 9) and counted via HTSeq (10).

### Gene Set Variation Analysis (GSVA)

The open source GSVA (V1.25.0) software package for R/Bioconductor (11) was used as a non-parametric, unsupervised method for estimating the variation of pre-defined gene sets in patient and control samples of microarray expression data sets. Raw RNAseq counts transformed into log2 expression values for pre-defined gene sets were used as the inputs for GSVA. Enrichment scores (GSVA scores) were calculated non-parametrically using a Kolmogorov Smirnoff (KS)-like random walk statistic; a negative value for a particular sample and gene set indicated that the gene set has a lower expression than the same gene set in a sample with a positive value. The enrichment scores (ES) were the largest positive and negative random walk deviations from zero, respectively, for a particular sample and gene set. The positive and negative ES for a particular gene set depend on the expression levels of the genes that form the pre-defined gene set. The increased transcripts for SLE plasma cells (PC) were taken from Lugar et al. (12) to determine the enrichment of PC. Tfh cells were determined by expression of *Bcl6* (13), *Pdcd1, Icos, Ascl2* (14), and *Tnfsf4* (15). Markers of germinal centers were determined by expression of *Gcsam* (16), *Nuggc* (17), *Rgs13* (18), *Klhl6* (19), *Aicda* (20), *Bcl6* (13), and *Irf4* (21).

### I-Scope Analysis

I-scope is a tool used to identify immune infiltrates in gene expression datasets. I-scope was created through an iterative search of more than 17,000 genes identified in more than 50 microarray datasets. From this search, 1226 candidate genes were identified and researched for restriction in hematopoietic cells as determined by the HPA, GTEx, and FANTOM5 datasets (www.proteinatlas.org) (22); 926 genes met the criteria for being mainly restricted to hematopoietic lineages (brain, reproductive organs exclusions). These genes were researched for immune cell specific expression in 30 hematopoietic sub-categories: T cells, regulatory T cells, activated T cells, anergic cells, CD4 T cells, CD8 T cells, gamma- delta T cells, NK/NKT cells, T & B cells, B cells, activated B cells, T &B & monocytes, monocytes & B cells, MHC Class II expressing cells, monocyte dendritic cells, dendritic cells, plasmacytoid dendritic cells, Langerhans cells, myeloid cells, plasma cells, erythrocytes, neutrophils, low density granulocytes, granulocytes, platelets, and all hematopoietic stem cells. Transcripts are entered into I-scope and the number of transcripts in each category are calculated and represent the specific immune cell populations in each dataset.

### Pathways Analysis

Ingenuity Pathway Analysis (IPA) software (Qiagen, Venlo, Netherlands) was used to calculate Z scores based on increased and decreased transcript levels in HDAC6 inhibitor samples compared with transcript levels in controls) (23). Z scores ≥ 2 or ≤ −2 and overlap *p*-values ≤ 0.05 were considered significant. IPA scores were used to determine whether pathways were up-regulated or repressed based on whether transcripts were increased or decreased relative to controls in the entry dataset.

### Gene Ontology (GO) Biological Pathway (BP) Analysis

Increased and decreased transcripts were annotated with GO BP terms separately and overlap *p*-values were determined. Pathways were considered enriched or reduced if they had associated *p*-values <0.01.

### Biologically Informed Gene Glustering (BIG-C)

BIG-C is a custom functional clustering tool developed to annotate the biological meaning of large lists of genes. Separately, increased and decreased genes are sorted into 52 categories based on their most likely biological function and/or cellular localization based on information from multiple online tools and databases including UniProtKB/Swiss-Prot, GO Terms, MGI database, KEGG pathways, NCBI PubMed, and the Interactome. Each gene is placed into only one category based on its most likely function to eliminate the redundancy in enrichment sometimes found in GO BP annotation (24, 25).

### Comparison of HDAC6 Inhibitor- Treated NZB/W RNA Seq to Human SLE Tissue Microarray Data

The comparison analysis feature of IPA (23) was used to compare the Z scores between processed microarray data from DE analysis of four human SLE tissue experiments and the DE analysis of the HDAC6 inhibitor treated vs. untreated NZB/W mice. Raw data from lupus tissue datasets were obtained from the GEO repository: GSE36700 for lupus synovium (4 OA, 4 SLE patients), GSE72535 for discoid lupus skin (8 healthy control (HC), 9 DLE), GSE32591 for LN dissected glomerulus WHO class 3 or 4 (32 HC, 22 SLE) and GSE32591 for lupus nephritis dissected tubulointerstitium from WHO CLASS 3 or 4 LN (32 HC, 22 SLE). Differential Gene Expression (DE) was carried out for each dataset of SLE tissue samples and controls. GCRMA normalized expression values were variance corrected using local empirical Bayesian shrinkage before calculation of DE using the ebayes function in the open source BioConductor LIMMA package (https://www.bioconductor.org/packages/release/bioc/html/limma.html) (26). Resulting *p*-values were adjusted for multiple hypothesis testing and filtered to retain DE probes with an FDR < 0.05 (27).

### Metabolic Enzyme Function Studies

Citrate synthase (CS) catalyzes the formation of citrate and coenzyme A (CoASH) from acetyl-CoA and oxaloacetate. CoASH reduces DTNB and CS activity was determined from the reduction of DTMB over time (28). Briefly, at sacrifice splenocytes from ACY-738 treated and control mice were lysed (1 × 10^6^ cells/200 μl) in a buffer containing 0.1% Triton X-100, 1 mM EDTA, 50 mM Tris, pH 7.4, and Protease Inhibitor Cocktail (Nacalai Tesque). The CS assay was carried out using 20 μl of the lysates in 96-well plates. CS activity was measured by adding 80 μl of the reaction solution containing 0.1 mM DTNB, 0.3 mM acetyl-CoA, 1 mM oxaloacetate, and 50 mM Tris at pH 7.4 to each well. Absorbance was measured on a spectrophotometer (BioTek Synergy 2, Winooski Vermont, USA) at 405 nm at 37C every 12 s for 5 min. Total protein concentration of the lysates was quantified by a Bio- Rad Protein Assay, and CS activity was normalized to the total protein concentration. CS activity was calculated as the rate of increase of absorbance with time. All samples were run in triplicate. Maximum activity was calculated and reported as μmol/mg/min.

For the determination of β-hydroxyacyl-CoA dehydrogenase activity, the oxidation of NADH to NAD was measured (28, 29). In this procedure, splenocytes were added to 190 μl of a buffer containing 0.1 M liquid triethanolamine, 5 mM EDTA tetrasodium salt dihydrate, and 0.45 mM NADH. Following a 2-min background reading, 15 μl of 2 mM acetoacetyl CoA was added to initiate the reaction. Absorbance was measured at 340 nm every 12 s for 5 min at 37C. Maximum activity was calculated and reported as μmol/mg/min.

Cytochrome c oxidase, which transfers electrons between complex III and IV of the electron transport chain, was assayed based on the oxidation of ferrocytochrome c to ferricytochrome c by cytochrome c oxidase. Horse heart cytochrome c (Sigma Aldrich, 2 mg/ml) was dissolved in a 10 mM potassium phosphate buffer containing 10 mg/ml of sodium dithionite. 10 ul of splenocyte extracts were added to 290 ul of the reduced cytochrome c test solution. The rate of cytochrome C oxidation was measured spectrophotometrically as a reduction in absorbance at 550 nm every 10 s for 5 min at 37C. Maximum cytochrome c oxidase activity was expressed relative to protein content and reported as μmol/mg/min.

### Fatty Acid and Glucose Oxidation Studies

Splenocytes were isolated from spleens from 8-week-old NZB/W female mice. T cells and B cells were enriched from splenocytes using negative selection with a magnetic-activated cell sorting kit (Miltenyi Biotec, Auburn CA). Cells were seeded in a 24-well flat- bottomed plate at a density of 1 × 10^6^ cells/ml in 1 ml RPMI-1640 (HyClone, South Logan, UT) supplemented with 1 mm sodium pyruvate, 2 mm l-glutamine, 100 U/ml penicillin, 100 μg/ml streptomycin (HyClone), 5·5 × 10^−2^ mm 2-mercaptoethanol (Gibco BRL Life Technologies, Paisley, UK) and 10% heat-inactivated bovine calf serum (HyClone) per well. For T cells stimulation, plates were pre-coated with anti-CD3 (Invitrogen) and T cells were stimulated with anti-CD28 (Invitrogen) with or without the addition of 4 uM ACY-738 or DMSO (control) followed by 24 h incubation at 37°C with 5% CO_2_. B cells were cultured with Lipopolysaccharide (LPS: *Escherichia coli* serotype 0111:B4; Sigma-Aldrich, St. Louis, MO) (50 ug/ml) and treated with ACY-738 (4 uM) or DMSO (control) for 24 h after which the cells were collected and metabolism analysis performed. Substrate metabolism was assessed as previously described (28). Briefly, fatty acid oxidation was measured using radiolabeled fatty acid ([1-^14^C]-palmitic acid, American Radiolabeled Chemicals, St. Louis, MO) to quantify ^14^CO_2_ production from the oxidation of isolated B and T cells. Cells were incubated in 0.5 μCi/mL of [1-^14^C]-palmitic acid for 1 h after which the media was acidified with 200 μL 45% perchloric acid for 1 h to liberate ^14^CO_2_. The ^14^CO_2_ was trapped in a tube containing 1 M NaOH, which was then placed into a scintillation vial with 5 mL scintillation fluid. The vial's ^14^C concentrations were measured on a 4500 Beckman Coulter scintillation counter. Glucose oxidation was assessed in the same manner as fatty acid oxidation with the exception that [U-^14^C] glucose was substituted for [1-^14^C]-palmitic acid. Oxidation values were normalized to total protein content as assessed via a commercially available bicinchroninic acid (bca) procedure (Thermo Fisher Scientific, Waltham, MA) and expressed as nM/mg protein/hr.

### Statistics

Data was analyzed by student *t*-test with GraphPad Prism software. Statistically significant differences are followed by ^*^P ≤ 0.05, ^**^P ≤ 0.01, ^***^P ≤ 0.001; ^****^P ≤ 0.0001.

### Study Approval

The animal experiments strictly followed the requirement of the Institutional Animal Care and Use Committee (IACUC) at Virginia Tech, USA and maintained under specific pathogen-free conditions at Virginia Tech College of Veterinary Medicine. All of operations of animals were in compliance with the Guide for the Care and Use of Laboratory Animals.

## Results

### ACY-738 Is Selective for HDAC6 Inhibition

**Figure 1:**
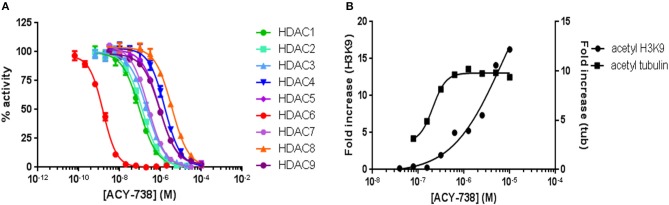
ACY-738 is a selective inhibitor or HDAC6. ACY-738 inhibits HDAC6 with a potency of 1.5 nM and HDAC1 (the next most affected target) with a potency of 93 nM **(A)**. In a cell based assay in HCT-116 cells ACY-738 Induces tubulin acetylation (a marker of HDAC6 inhibition) at 800 nM, whereas histone acetylation (a marker of Class 1 HDAC inhibition) at that concentration is minimal **(B)**.

ACY-738 is a hydroxamic acid HDAC6 inhibitor that is highly selective for HDAC6. In preliminary experiments, we found that ACY-738 inhibits HDAC6 with a potency of 1.5 nM and HDAC1 (the next most affected target) with a potency of 93 nM (Figure 1A). In a cell-based assay using HCT-116 cells, we found ACY-738 induced tubulin acetylation (a marker of HDAC6 inhibition) at 800 nM, whereas acetylation of H3K9 (a marker of Class 1 HDAC inhibition) at that concentration was minimal suggesting that the inhibition was primarily cytosolic (Figure 1B). As previously reported, 100 mg/kg/day of ACY-738 in rodent chow achieves an estimated plasma concentration of 100 nM (30). In our studies, the mean plasma concentration of ACY-738 at different time intervals was 212 nM as determined by mass spectrometry (LC/MS, Agilux, Worcester, MA, USA).

### Inhibition of HDAC6 Improves Established LN

**Figure 2:**
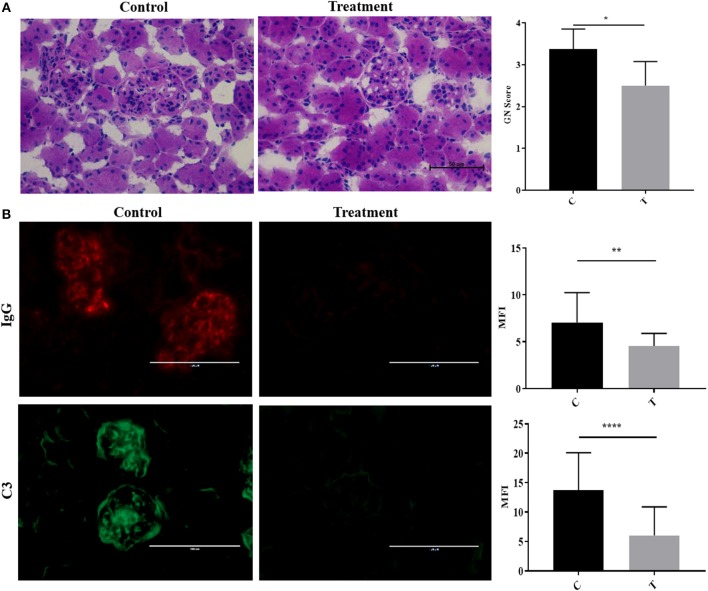
Inhibition of histone deacetylase HDAC6 reduced Ig and C deposition in NZB/W lupus nephritis. **(A)** Representative Hematoxylin and Eosin (H&E) staining image of kidney glomerular region along with pathology score which reflects the severity of membranoproliferative changes and distribution. **(B)** Representative immunohistological staining of kidney section for IgG and C3. Graphic analysis of mean fluorescent intensity (MFI) of IgG and C3 is also provided. Data are shown as mean ± standard error of the mean (s.e.m) *n* = 4 mice for each group; *T*-test; ^*^P < 0.05, ^**^P < 0.01, ^****^P < 0.0001.

To simulate the therapeutic paradigm in human lupus, we treated 20-week-old NZB/W F1 female (NZB/W) mice with established LN with the selective HDAC6 inhibitor ACY-738. After only 4 weeks, ACY-738-treated mice exhibited significantly less renal pathology than the untreated group (Figure 2A). Moreover, the deposition of IgG and C3 in glomeruli, which contribute to the progression of renal inflammation, was significantly decreased in the ACY-738 treated group compared to the untreated control group (Figure 2B).

### Suppression of B Cell Responses by HDAC6 Inhibitor

**Figure 3:**
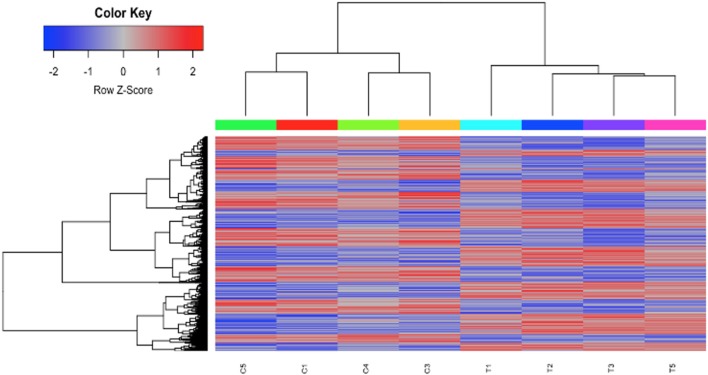
HDAC6i treatment of NZB/NZW F1 mice induced global gene expression changes in whole splenocytes. Hierarchical clustering of 3911 transcripts (1922 up, 1989 down) that differed significantly (FDR < 0.1) between control (C1, C3, C4, and C5) and treated mice (T1, T2, T3, and T5).

**Figure 4:**
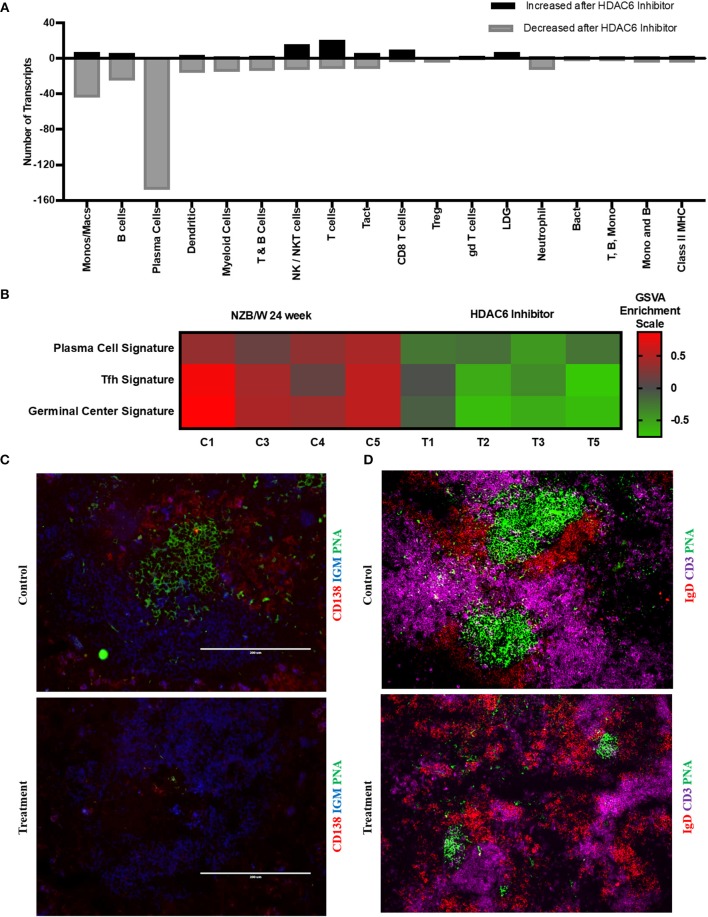
HDAC6i treatment results in significantly decreased GC activity and PC formation. **(A)** I-Scope hematopoietic cell enrichment demonstrated that HDAC6 inhibition decreased PC, B cells and inflammatory myeloid cells. The number of transcripts corresponding to each cell type increased or decreased after HDAC6 inhibitor treatment are shown. Gene symbols for transcripts for PC, B cells and inflammatory myeloid cell are in Supplementary Data 1 (increased transcripts) and Supplementary Data 2 (decreased transcripts). **(B)** GSVA was carried out to determine the enrichment of PC, Tfh cells and GC in each HDAC6 inhibitor treated and control NZB/NZW mouse (Methods lists genes used for GSVA enrichment modules). **(C)** Representative splenic section stained with anti-CD138, anti-IgM and PNA. **(D)** Representative splenic section stained for T cells, follicular B cells and GC with anti- CD3, anti-IgD, and PNA.

To investigate the mechanisms of HDAC6 inhibition on autoimmune responses, we analyzed changes in splenic composition by carrying out bulk RNA sequencing on total splenocytes from ACY-738-treated and untreated NZB/W mice (Figure 3). Analysis of global gene expression changes by hierarchical clustering showed that 3911 transcripts were differentially expressed between the treated and untreated samples. Among these, 1922 genes were up-regulated, and 1989 genes were down-regulated in the ACY-738-treated group compared to the control group. To determine whether HDAC6 inhibition led to changes in cell populations in the spleen of treated mice, we employed the I-Scope clustering program that permitted identification of immune and inflammatory cell types based on gene expression. Control experiments were performed to demonstrate the specificity and lack of cross reactivity of I-scope (Supplementary Figures 5A,B). We found that HDAC6 inhibition led to a profound decrease in transcripts associated with plasma cells, B cells and inflammatory myeloid cells (Figure 4A and Supplementary Datas 1, 2) as well as more modest decreases in other immune/inflammatory cells. Next, gene set variation analysis (GSVA) was carried out to determine whether there was enrichment in transcripts identifying these populations. Indeed, we found that plasma cell, Tfh cell, and GC signatures were all decreased following 4 weeks of HDAC6 inhibitor treatment, as compared to the untreated control (Figure 4B). To validate further the impact of the HDAC6 inhibitor on germinal center B cell response, we assessed the changes of spleens and Peyer's patches from C57BL/6J/HDAC6^−/−^ mice compared to C57BL/6J mice by flow cytometry (Supplementary Figures 2A,B). We found a reduction of T follicular helper cells (Tfh) in spleens and Peyer's patches of HDAC6 knockout mice compared to wild type C57BL/6J mice. Different from lupus-prone mice, the lack of HDAC6 in mice of B6 background showed no reduction of splenic spontaneous germinal centers in steady state. This suggests that there are differences in molecular pathways in splenic germinal center formation in lupus mice compared to non-lupus prone mice. To confirm our RNA sequencing results, we carried out immunohistofluorescence (IHF) microscopy of splenic sections to evaluate the presence of plasma cells and GCs (Figures 4C,D). Consistent with the RNA sequencing results, both CD138^+^ PC (Figure 4C and Supplementary Figure 1B) and PNA^+^ GC (Figure 4D and Supplementary Figure 1A) were dramatically reduced in the ACY-738-treated group, suggesting that HDAC6 treatment suppressed GC activity and subsequent PC generation and/or survival.

### HDAC6 Inhibition Reduces B Cell Signaling in NZB/NZW F1 Mice

**Figure 5:**
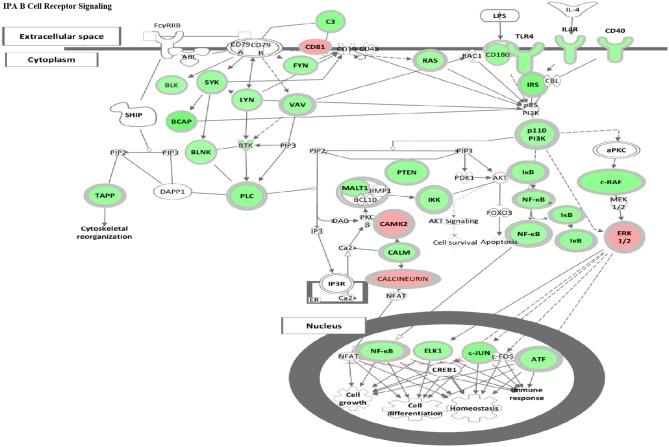
HDAC6 inhibition repressed B cell signaling pathways in NZB/NZW mice. The IPA Canonical Signaling Pathway “B Cell Receptor Signaling” had a Z score of −3.1. Transcripts differentially expressed between HDAC6 inhibitor treated and untreated NZB/NZW mice were overlaid on genes in the IPA pathway. Decreased transcripts are shown in green, while increased transcripts are shown in pink.

To evaluate whether HDAC6 inhibition specifically might inhibit B cell signaling, we employed IPA canonical pathway analysis to assess the pattern of change in differential gene expression in HDAC6-treated mice (Figure 5). HDAC6 inhibition was found to reduce transcripts involved in both the BCR and the TLR dependent PI3K signaling pathway in B cells, as well as decreasing transcription factors, NF-κB, ELK1, c-JUN and ATF, which control cell growth, differentiation, and homeostasis of many cells including B cells. To validate the role of HDAC6 in regulation of B cell activation signaling suggested by analysis of RNAseq data, we have carried out *in vitro* stimulation experiments with HDAC6^−/−^ mice and NZB/W mice (Figure 4 and Supplementary Figures 3, 4). We found reduced activation of B cells in B cells from C57BL/6J/HDAC6^−/−^ mice as well as ACY-738 treated NZB/W mice.

### HDAC6 Inhibition Alters Gene Transcripts Associated With Inflammation and Cellular Metabolism

**Figure 6:**
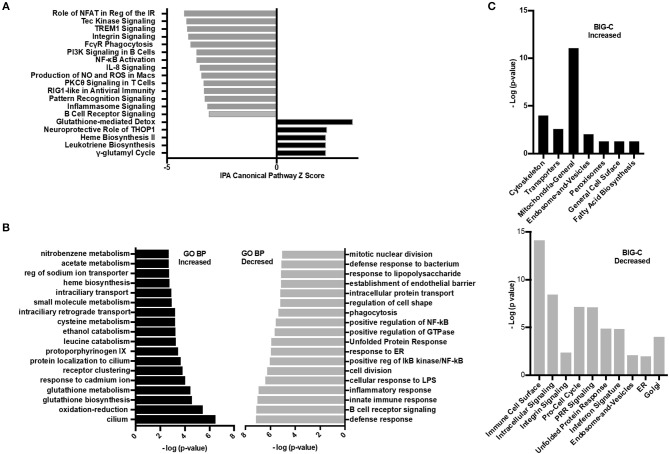
Inhibition of HDAC6 altered transcripts associated with cellular metabolism. **(A)** Ingenuity pathway analysis (IPA) was performed on the differentially expressed transcripts between HDAC6 inhibitor treated and untreated NZB/NZW mice. The most significant signaling pathways increased or decreased by Z score analysis with an overlap *p*-value ≤ 0.05 are shown. The full list of significant increased and decreased pathways and the genes used to determine significance are in Supplementary Data 3 (increased) and Supplementary Data 4 (decreased). **(B)** GO biological pathway enrichment analysis of the top most increased and decreased pathways by lowest overlap *p*-value significance. A full list of GO biological pathways enriched (*p* < 0.01) are in Supplementary Data 5 (increased) and Supplementary Data 6 (decreased). **(C)** BIG-C pathway enrichment was performed using increased (left) or decreased (right) transcripts from the DE analysis of HDAC6 inhibitor treated NZB/NZW mice compared to NZB/NZW mice. The -log (*p*-value) is shown for the enriched categories. Gene symbols corresponding to each category are listed in Supplementary Data 7 (increased) and Supplementary Data 8 (decreased).

To investigate further the specific pathways by which HDAC6 inhibition decreased the molecular basis of lupus, several additional analyses were carried out (Figure 6). IPA was used to determine the biological pathways significantly affected by HDAC6 inhibitor treatment (Figure 6A). There were only five significant signaling pathways increased by HDAC6 inhibitor treatment (*p* ≤ 0.05; Z ≥ 2). We found that HDAC6 inhibition led to an increase in glutathione metabolism and the gamma-glutamyl cycle, which may be related to the activation of the mercapturic acid pathway for the detoxification of foreign compounds. With regard to pathways down-regulated with HDAC6 inhibition, there were 59 pathways with Z scores ≤ −2 and *p*-values ≤ 0.05; pathways associated with immune signaling, B cell signaling, myeloid inflammatory pathways and phagocytosis—all pathways previously demonstrated to be important in the pathogenesis of human SLE (31–33). Next, GO biological pathway enrichment analysis was carried out separately on increased and decreased transcripts and categories with significant overlap *p*-values were determined (Figure 6B). GO biological pathway analysis confirmed the increase in biochemical processes associated with drug metabolism shown by IPA, but processes related to cilium assembly were most highly enriched. The most decreased GO categories were related to the immune and inflammatory response, B cell receptor signaling, cell division, ER stress and unfolded protein responses, NF-κB signaling and phagocytosis. Furthermore, a decrease in the interferon gene signature as well as pattern recognition receptors such as TLRs was also observed. These results illustrate that the IPA pathways and the GO biological pathway analysis showed similar changes in transcription and signaling profiles.

Next, the enrichment of transcripts increased or decreased in HDAC6 treated NZB/NZW mice were assessed using the BIG-C clustering algorithm and chi square analysis to evaluate significant enrichment of BIG-C categories (Figure 6C). In agreement with published data from human patients treated with HDAC inhibitors, we observed a significant metabolic shift as evidenced by the increase in biochemical markers in the cytoplasm, including enzymes associated with fatty acid synthesis, mitochondrial and peroxisome activity (34) (Supplementary Data 7). Furthermore, the observed decrease in transcripts associated with the unfolded protein response, golgi, ER, and cell cycle transcripts associated with recently generated plasma cells support an overall reduction in plasma cells (Supplementary Data 8). Thus, the three analytical methodologies demonstrated that HDAC6 inhibitor treatment led to increased transcripts associated with biochemical pathways and cytoskeletal events and decreased transcripts associated with plasma cells and immune networks.

### HDAC6 Inhibition Alters Cellular Metabolism

**Figure 7:**
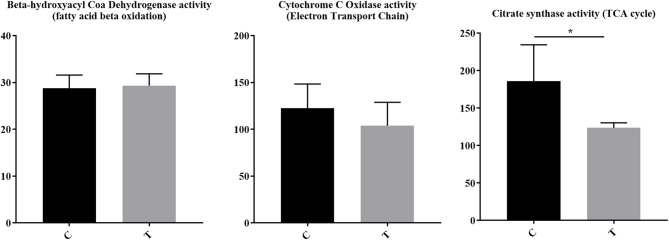
HDAC6 inhibition decreased citrate synthase activity and cytochrome c oxidase activity in NZB/W mice. Four weeks of treatment of NZB/W mice with the HDAC6 inhibitor ACY-738 lead to a significant decrease in the rate limiting enzyme of the TCA cycle (*p* = 0.043), and a decrease in cytochrome C oxidase activity (*p* = 0.053) while having no effect on beta hydroxyacyl coa dehydrogenase in splenocytes (*n* = 5). ^*^P < 0.05.

For immune cells to become activated, metabolic processes increase to support activation, proliferation, and differentiation. Although pathways associated with the mitochondria and cellular biochemistry were affected by HDAC6 inhibition, it was unclear whether a specific type of metabolism was predominating after treatment. Increased transcripts related to cellular energy production demonstrated nine genes associated with glycolysis (*Fbp1* (negative regulator), *Ier3* (negative regulator), *G6pc3, Pfkm, Aldoc, Dhktd1, Prkaa2, Khk, Eno2*), 12 genes involved in oxidative phosphorylation (*Taz, Atp5s, Slc25a23, Cox4l2, Cox6b2, Ndufb3, mt-Nd2, mt-Nd4, mt-Cytb, Nipsap2, Coq7*, and *Nubpl*), seven fatty acid beta-oxidation genes (*Acsbg1, Slc27a6, Slc27a1, Ivd, Pex5, Pex7, Hadh, Decr1, Echdc2, Acad11*) and four genes associated with the TCA cycle (*Pdk2* (negative regulator), *Idh2, Sdhaf4, Dhtkd1*). Among decreased transcripts, there were nine genes associated with glycolysis (*Pgk1, Pgam1, Pfkfb3, Hk2, Pfkp* (expressed in platelets and fibroblasts), *Zbtb7a, Nupr1, Hif1a, Tpil1*), seven with oxidative phosphorylation (*Coa5, Nupr1, Pgk1, Atp7a, Bid, Vcp, Pde12*), two with fatty acid beta oxidation (*Abcd1, Abcd2*), and four with the TCA cycle (*Glud1, Idh1, Pdha1, Pdpr*). To determine whether the altered transcripts induced by HDAC inhibition led to altered metabolic pathways in lupus mice, we examined the enzyme activity of proteins involved in electron transport chain function, the tricarboxylic acid cycle, and fatty acid beta oxidation in the spleens of lupus mice treated with the HDAC6 inhibitor ACY-728 for a 4-week period (Figure 7). We observed a significant decline in citrate synthase enzyme function in response to HDAC inhibition (*p* = 0.043). The activity of citrate synthase is a biochemical marker of mitochondrial density and oxidative capacity (35, 36). The activities of beta hydroxyacyl CoA dehydrogenase (βHAD), a key regulatory enzyme in the beta oxidation of fatty acids to acetyl CoA was unchanged with HDAC6 inhibition whereas cytochrome c oxidase, important in the function of mitochondrial electron transport chain function, was decreased but not statistically significant (*p* = 0.053).

**Figure 8:**
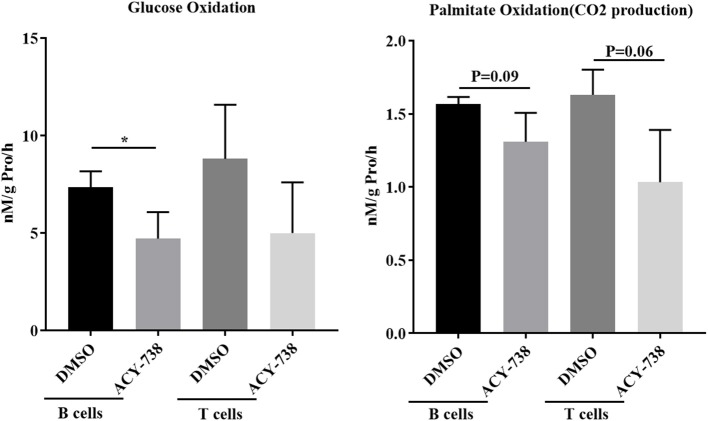
HDAC6 inhibition decreases glucose and fatty acid oxidation in T and B cells from NZB/W mice. T cells and B cells from 12-week old NZB/W female were purified and stimulated with anti CD3/CD28 or LPS, respectively, for 24 h with or without the addition of 4 uM ACY-738 (DMSO only was used as control). After 24 h of culture, CO_2_ production from the oxidation of glucose and palmitate were determined from three separate experiments in triplicate (*n* = 3). ^*^P < 0.05.

To investigate further the role of ACY-738 on the metabolic function of B and T cells, we performed *in vitro* experiments on cells isolated from NZB/W female lupus mice. Purified B cells and T cells were stimulated with LPS or anti CD3/CD28 for 24 h with or without 4 uM of ACY-738 (Figure 8). We have previously reported that at this concentration of ACY-738 is effective at inhibiting inflammatory mediator production and activation in immune cells without toxicity (37). Glucose is a major source for energy and biosynthesis in activated T and B cells (38, 39). In the cell, glucose undergoes a 10-step reaction to generate pyruvate which is either reduced into lactate by lactate dehydrogenase in the cytosol, or transported into the mitochondria via the mitochondrial pyruvate carrier complex where it is converted into acetyl-CoA by the pyruvate dehydrogenase complex, a process that is tightly regulated by the pyruvate dehydrogenase kinase (*Pdk1*), which can phosphorylate pyruvate dehydrogenase complex and inhibit its activity. When B cells were treated with ACY-738, CO_2_ produced from oxidation of glucose was significantly decreased (*p* = 0.044). In T cells there was a reduction in CO_2_ after treatment, but it was not significant (*p* = 0.16) (Figure 8). Next, we investigated the amount of CO_2_ production from fatty acids (palmitate) with and without ACY-738. Similarly, we found that ACY-738 did not decrease CO_2_ production from fatty acids in stimulated B cell and T cells significantly (*P* = 0.09, B cells and *P* = 0.06, T cells).

### HDAC6 Inhibition in Mice Decreases Pathogenic Signaling Pathways That Are Up-Regulated in Active Human SLE

**Figure 9:**
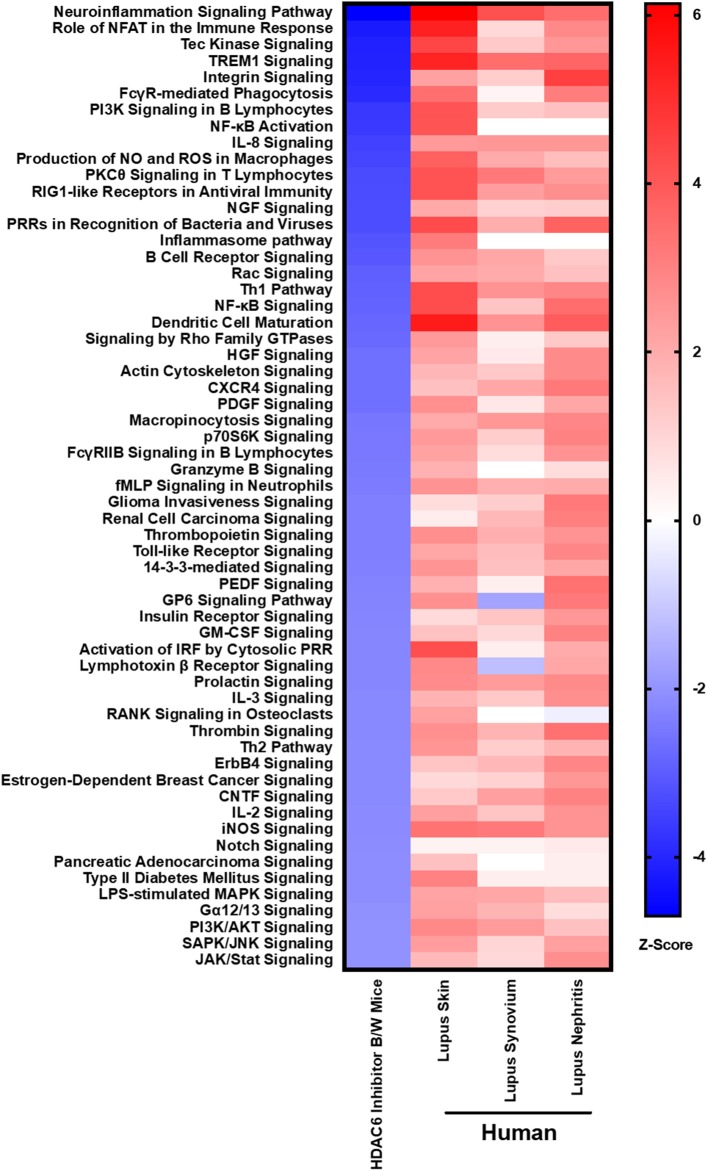
HDAC6 inhibition decreases lupus gene signature pathways in NZB/W mice that are increased in active human SLE. IPA canonical signaling pathways increased in human SLE microarray tissue datasets were compared to signaling pathways in NZB/W mice decreased by the HDAC6 inhibitor. Z scores greater or <2 are considered significant.

In order to demonstrate the relevance of our findings regarding HDAC6 inhibitor-mediated suppression of molecular pathways in lupus mice, we compared the downregulated pathways to those found to be up-regulated in active human lupus. Specifically, we compared the pathways down-regulated by HDAC6 inhibition in NZB/W mice to pathways up-regulated in human lupus affected organs, including skin, synovium and kidney (Figure 9). Our results showed that the molecular pathways decreased by the HDAC6 inhibitor in NZB/W mice are also highly up-regulated in human SLE affected tissues. For example, ACY-738 treatment of NZB/W mice significantly decreased a total of 59 IPA canonical pathways (Z ≤ −2, *p* ≤ 0.05). Of these pathways, 38 (64%) had significant positive Z scores (Z > 2) for at least two of three human SLE affected tissues. For the remaining 21 IPA canonical pathways decreased by ACY-738, positive Z scores <2 were found for most of the human SLE affected tissues. ACY-738 treatment of mice increased a total of 5 canonical pathways, and none were significantly decreased in human SLE although glutathione mediated detoxification (Z = 3.5 in HDAC6 inhibitor treated mice) had negative Z scores for human lupus skin (−1.8) and lupus nephritis (−1.6). The striking overlap in canonical pathways affected by HDAC6 inhibition and the aberrant pathways in human SLE affected tissues confirms the relevance of the murine lupus results in predicting potential benefit in human lupus.

## Discussion

In a previous study (40), we found that the expression of HDAC6 and its activity was significantly increased in B cells from lupus prone mice compared to healthy control mice, suggesting the pathogenic role of HDAC6 in lupus. In the current studies, we sought to define the mechanism by which HDAC6 inhibition decreases disease pathogenesis in NZB/W mice by using RNAseq to evaluate the transcriptomic signatures of splenocytes from treated and untreated mice coupled with applied computational cellular and pathway analysis. In addition, we sought to bridge between the transcriptomic data obtained from the HDAC6 treated mice and human gene expression information to determine the relevance to this target in possibly controlling human lupus. We found that PC development was abrogated and GC formation was greatly reduced in HDAC6 inhibitor-treted NZB/W mice. When we compared the HDAC6 inhibitor treated lupus mouse gene signatures to human lupus patient gene signatures, the results showed numerous immune and inflammatory pathways increased in active human lupus affected tissue were significantly decreased in the HDAC6 inhibitor treated animals. Pathway analysis suggested alterations in cellular metabolism might contribute to the normalization of lupus mouse spleen genomic signatures, and this was confirmed by direct measurement of the impact of the HDAC6 inhibitor on metabolic activities of murine spleen cells. Taken together, our studies suggest that HDAC6 may decrease germinal center activity and B cell activation and reduces several signaling pathways required for PC differentiation in the context of LN. Moreover, the molecular pathways suppressed by the HDAC6 inhibitor were frequently overexpressed in human lupus tissue. Of importance, our data also suggests that HDAC6 inhibition corrects aberrant cellular metabolism observed in lupus.

There are numerous signaling pathways, metabolic events, and transcription factors that regulate the differentiation of B cells into PC (41–45). Our rationale for continued investigations to define the molecular events in lupus immunopathogenesis mediated by HDAC6 is related to the uncertainty of the non-redundant roles of HDAC6 in immune function in general and lupus in particular. HDAC6 knock out mice (HDAC6^−/−^) have grossly normal immune cell development. However, HDAC6^−/−^ mice show a 4-fold decrease in antibody production in response to immunization with a T cell-dependent antigen. Furthermore, responses to RNA but not DNA viruses are reduced in HDAC6-deficient mice (46, 47). HDAC6 is a unique member of the HDAC family that largely resides within the cytoplasm and regulates the acetylation status of a number of cytoplasmic proteins. These include proteins involved in the tubulin cytoskeleton as well as the proteasome. HDAC6 inhibition, therefore, has the potential to alter a variety of cellular functions (48, 49). Inhibition of HDAC6 has also beneficial effects treating multiple myeloma, an expansion of malignant PCs that secrete abnormal antibodies (50, 51). In lupus, HDAC6 may act to regulate both innate and adaptive immune responses (52–56). HDAC6 acts as a coactivator for interferon-beta (IFN-β) induction, and HDAC6 inhibition prevents IFN-β expression (57). Indeed, we found that the IFN signature is decreased. β-catenin also serves a target of HDAC6; deacetylation of β-catenin facilitates it translocation to the nucleus to serve as a co-activator for IRF3-mediated transcription, a possible mechanism for its impact on IFN- β production (58). In B cells, HDAC6 inhibition leads to the acetylation of NFκB which prevents its nuclear translocation (59, 60). Alpha tubulin regulates the cellular cytoskeleton and is acetylated by HDAC6 inhibitors. Increased acetylation of alpha tubulin may inhibit the B-T cell interaction by preventing B cell migration and germinal center formation. Indeed, Tfh-B cell collaboration requires interaction of CD40L and IL-4 with CD40 and IL-4L, respectively. We have previously shown that HDAC6 inhibition resulted in a decreased Tfh population and reduced CD40 and IL-4L activities in B cells (37). The current studies confirm that HDAC6 inhibition decreased the Tfh population in lupus mice. Additionally, regulation of B cell activation involves tyrosine kinase regulation. P85/P110-PI3K belongs to class IA PI3K mediated signals which regulate B cell commitment and differentiation. PI3K signaling pathways can be activated in a Toll like receptor (TLC)-dependent or B cell receptor (BCR)-dependent manner. Following treatment with ACY-738, we found decreased PI3K transcripts which are important for B cell inflammatory signaling (61). Bruton's tyrosine kinase (Btk) is also an important component of BCR signaling. Of note, increased Btk expression has been observed in human autoimmune disease (62). Prior studies have shown Bkt activation controls the entry of peripheral naïve B cells into the follicle, survival and maturation of B cells, and plasma cell differentiation (63). Recent studies have demonstrated that inhibition of Btk reduces autoantibody production and pathogenesis (64, 65). Btk inhibition reduces B cell activation, differentiation of PC and autoantibody class-switching (62). In our current studies, we found that Btk expression and signaling cascade was suppressed by HDAC6 inhibition, the suppression of Btk may have occurred through inhibition of PI3K signaling. In summary, the HDAC6 inhibitor suppresses expression of a number of pathways that are essential for B cell activation and differentiation of PC. Whether the therapeutic effect in SLE is based on inhibition of one pathway or many activation pathways required for germinal center formation and PC differentiation and survival remains to be completely defined.

HDAC6 inhibitor treatment was also demonstrated to have an effect on cellular metabolism. This was shown *in vivo* in treated mice and *in vitro* with cultured lymphocytes. In regard to cellular metabolism, we observed a significant metabolic shift as evidenced by the increase in gene expression profiles of biochemical markers in the cytoplasm, including mitochondrial enzymes associated with fatty acid oxidation and peroxisome activity which others have reported with HDAC6i (66, 67). Despite an increase in mRNA content of mitochondrial enzymes, we observed a significant decline in citrate synthase enzyme function in response to HDAC6 inhibition. The activity of citrate synthase is a biochemical marker of mitochondrial density and oxidative capacity (35, 36). Perhaps the increased gene expression signature is compensatory to a reduced enzyme activity. Indeed, it has been shown previously that mitochondrial metabolism, including citrate synthase activity, is downregulated in response to HDAC6 inhibition (68). This is an important finding as O_2_ consumption was found to be increased in SLE patents relative to control subjects (34). Furthermore, the electron transport chain complex I has been identified as the main source of oxidative stress in SLE (69). B cell differentiation to PC requires a terminal increase in oxidative phosphorylation in order to generate antibodies (70). The activities of beta hydroxyacyl coA dehydrogenase (βHAD), a key regulatory enzyme in the beta oxidation of fatty acids to acetyl CoA was unchanged with HDAC6 inhibition whereas cytochrome c oxidase, important in the function of the mitochondrial electron transport chain function was decreased but not significantly. Metabolic control of mitochondrial ROS production and glucose utilization have long been recognized as regulators of cellular activation within T cells (71). In particular, glucose utilization via the pentose phosphate pathway (PPP) and output of NADPH have been reported to regulate the mitochondrial transmembrane potential during T cell activation and chronic activation of CD4^+^ T cells from lupus-prone mice and SLE patients occurs with high levels of oxygen consumption (72). Indeed, in other immune-mediated inflammatory diseases, it has been reported that there is increased activation of the citric acid cycle is associated with disease (73). Taken together, these studies suggest that HDAC6 inhibition may decrease lupus disease by regulating immunologic as well as metabolic function.

To investigate further whether HDAC6 inhibition directly decreased cellular metabolism or whether the changes noted in treated animals were secondary to quieting of the immune response, we stimulated NZB/W B and T cells *in vitro* and with and without the HDAC6 inhibitor ACY-738 and found that glucose metabolism was significantly decreased in B cells and that fatty acid oxidation was also reduced with HDAC6 inhibition. Combining our gene expression studies along with our *in vitro* metabolic studies suggest that glucose metabolism is critical for immune cell activation and inflammatory cytokine production. A recent study of human CD4^+^ T cells showed upregulation in metabolism, including pyruvate oxidation and TCA cycle utilization (74), resulting in cell polarization and production of IFN-γ production. Our *in vitro* results suggest that ACY-738 may limit cell metabolism and decrease the spontaneous activation of lupus T and B cells.

In summary, we have shown that selective HDAC6 inhibition corrects abnormal B cell activation and differentiation in NZB/W mice that display early onset disease. The correction in B cell differentiation and activation correlated with less severe renal disease. Specifically, HDAC6 inhibition decreased several signaling pathways that are critical for B cells differentiation to PC. In addition to HDAC6 inhibiting B cell and T cell activation, several metabolic and enzymes pathways that are known to be increased in active lupus were also ameliorated. This was demonstrated *in vivo* and *in vitro*. Finally, when RNA profiles from the NZB/W mice were compared to humans with lupus, our results demonstrate that the many of genes upregulated in lupus patients were decreased lupus mice with HDAC6 inhibition. Taken together, these studies suggest that selective HDAC6 inhibition may be a potential therapeutic for the treatment of lupus nephrites.

## Data Availability Statement

R bioconductor packages limma and Gene set variation analysis (GSVA) are open source code available at www.bioconductor.org. All other datasets are included in the manuscript/Supplementary Files.

## Ethics Statement

This study was carried out in accordance with the recommendations of the Guide for the Care and Use of Laboratory Animals. The protocol was approved by the Institutional Animal Care and Use Committee (IACUC) at Virginia Tech, USA.

## Author Contributions

JR and MC designed and conducted experiments, analyzed data, and wrote the manuscript. KE, XLi, KR, XLu, RM, MH, and MJ conducted experiment and contribute to manuscript suggestion. PB and AG contributed to data analysis. PL and CR supervised all studies and the manuscript.

### Conflict of Interest

MC, PB, AG, and PL are employed by AMPEL Biolosolutions. MJ is employed by Regenacy Pharmaceuticals. The remaining authors declare that the research was conducted in the absence of any commercial or financial relationships that could be construed as a potential conflict of interest.

## Supplementary Material

Supplementary Figure 1 Quantified germinal center formation in NZB/W female mice at 24 weeks-of age-treated with (T) or without (C) ACY-738 for four weeks. We randomly picked 5 germinal centers from each spleen sample and analyzed by using Image J software to calculate the size of the germinal center. *N* = 20, ^*^*P* < 0.05, ^****^*P* < 0.0001.

Supplementary Figure 2 Flow cytometry of GC B cells and TFH assessed by Flow cytometry in C57/B6 mice and C57B6HDAC6^−/−^ mice. For spleen, *n* = 5, for Peyer's patch, *n* = 3. Germinal center B cells are gated by CD19+, GL7+, IgD−. ^*^*P* < 0.05.

Supplementary Figure 3 Flow cytometry of sorted B cells from C57BL/6J mice and C57BL/6J/HDAC6^−/−^ mice stimulated with LPS or anti-IgM, anti-CD40 for 24 h. The results showed reduced expression of activation markers of B cells CD86 and MHCII in C57BL/6J/HDAC6^−/−^ mice compared to C57BL/6J mice with stimulation of anti-IgM and anti-CD40. In addition, MFI of CD69, CD86, and CD80 are downregulated in C57BL/6J/HDAC6^−/−^ mice with stimulation of LPS. *N* = 5. ^*^*P* < 0.05, ^**^*P* < 0.01.

Supplementary Figure 4 Flow cytometry of sorted B cells from NZB/W mice stimulated with LPS or anti-IgM, anti-CD40 and then treated with ACY738 for 24 h. The results showed reduced expression of activation markers of B cells CD86 and MHCII in ACY-738 treated B cells with stimulation of anti-IgM and anti-CD40. In addition, MFI of CD69, CD86, MHC-II, and CD80 are significantly downregulated in ACY-738 treated B cells with stimulation of LPS. *N* = 5. ^*^*P* < 0.05, ^**^*P* < 0.01, ^***^*P* < 0.001, ^****^*P* < 0.0001.

Supplementary Figure 5 **(A)** Control experiments demonstrate the specificity and lack of cross reactivity of I-scope. Experiments were performed on the DE analysis of healthy control purified CD3+CD4+ T cells, CD19+CD3− B and Plasma Cells, and CD33+CD3− Myeloid cells from microarray dataset GSE10325. The genes in each I-scope category (29 categories in total; hematopoietic general was not used) were used as modules for gene set variation analysis to determine the specificity of each module and cross-reactivity to other cell types. For each comparison, only categories with at least three genes above the Interquartile Range threshold were considered for statistical analysis. Significance of GSVA enrichment scores was determined using Sidak's multiple comparisons test. Adjusted p-values below 0.05 were considered significant. **(B)** Demonstration of strong relationship of human B cell/microliter counts to GSVA enrichment scores for the I-scope B cell category on 105 human subjects from microarray dataset GSE88884. Demonstration of the strong relationship of mouse flow cytometry values for plasma cells (B220+IgM−CD138+) and the GSVA enrichment scores using the I-scope plasma cell module on BXSB Yaa and BXSB MPJ mice.
