# Supplementary figures and images for: Selective Histone Deacetylase 6 Inhibition Normalizes B Cell Activation and Germinal Center Formation in a Model of Systemic Lupus Erythematosus
Source: Front Immunol. 2019 Oct 25;10:2512. doi: 10.3389/fimmu.2019.02512 (PMC6823248; doi:10.3389/fimmu.2019.02512)

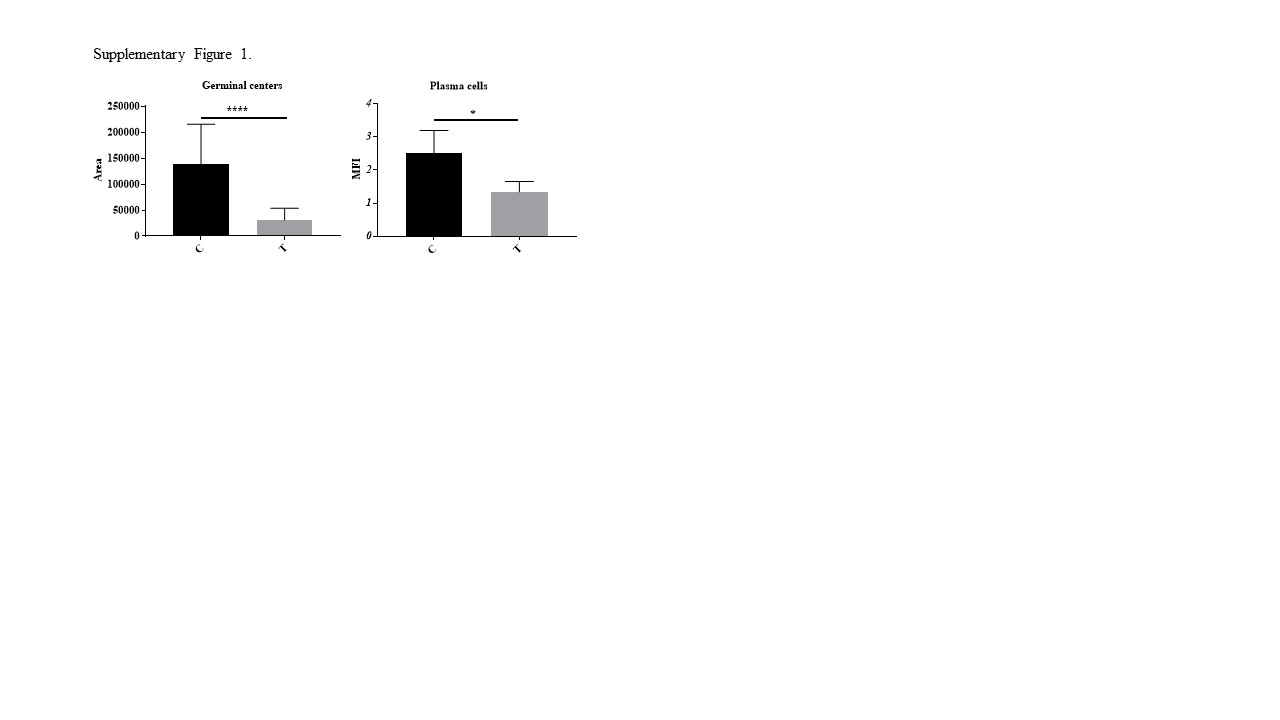

Supplement: Supplementary Figure 1 — Quantified germinal center formation in NZB/W female mice at 24 weeks-of age-treated with (T) or without (C) ACY-738 for four weeks. We randomly picked 5 germinal centers from each spleen sample and analyzed by using Image J software to calculate the size of the germinal center. N = 20, *P < 0.05, ****P < 0.0001. [file Image_1.jpg]

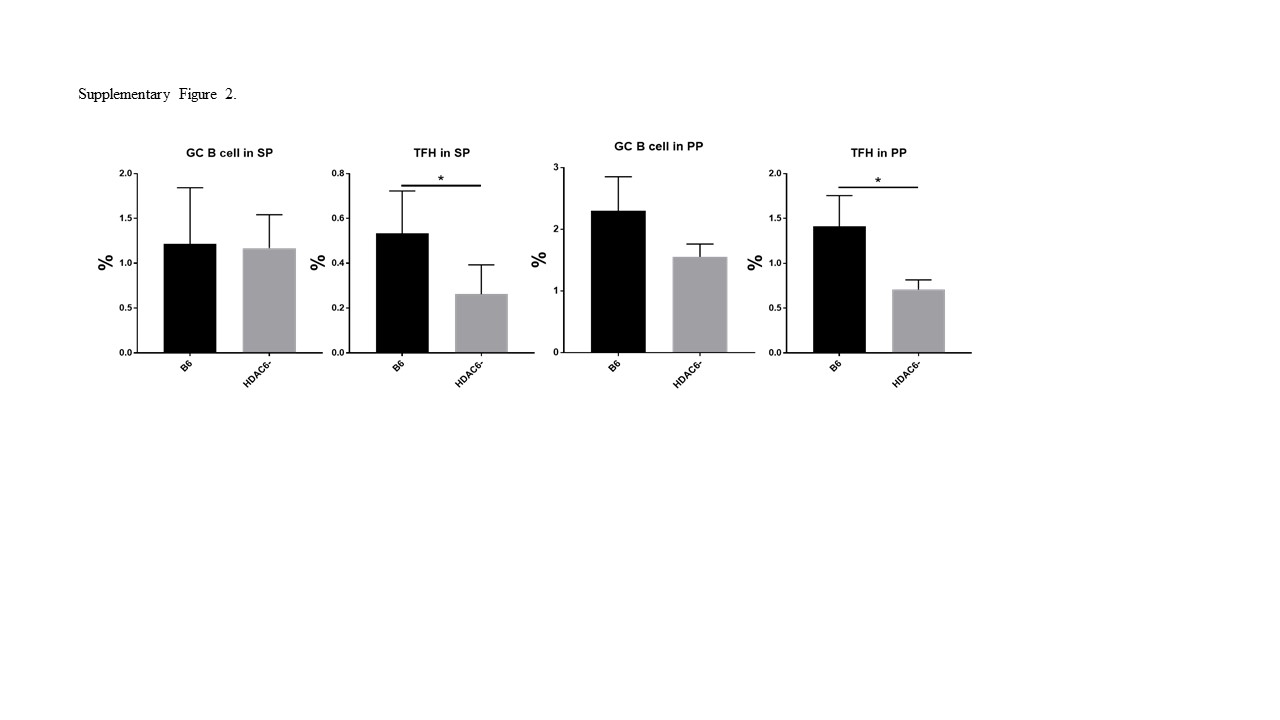

Supplement: Supplementary Figure 2 — Flow cytometry of GC B cells and TFH assessed by Flow cytometry in C57/B6 mice and C57B6HDAC6−/− mice. For spleen, n = 5, for Peyer's patch, n = 3. Germinal center B cells are gated by CD19+, GL7+, IgD−. *P < 0.05. [file Image_2.jpg]

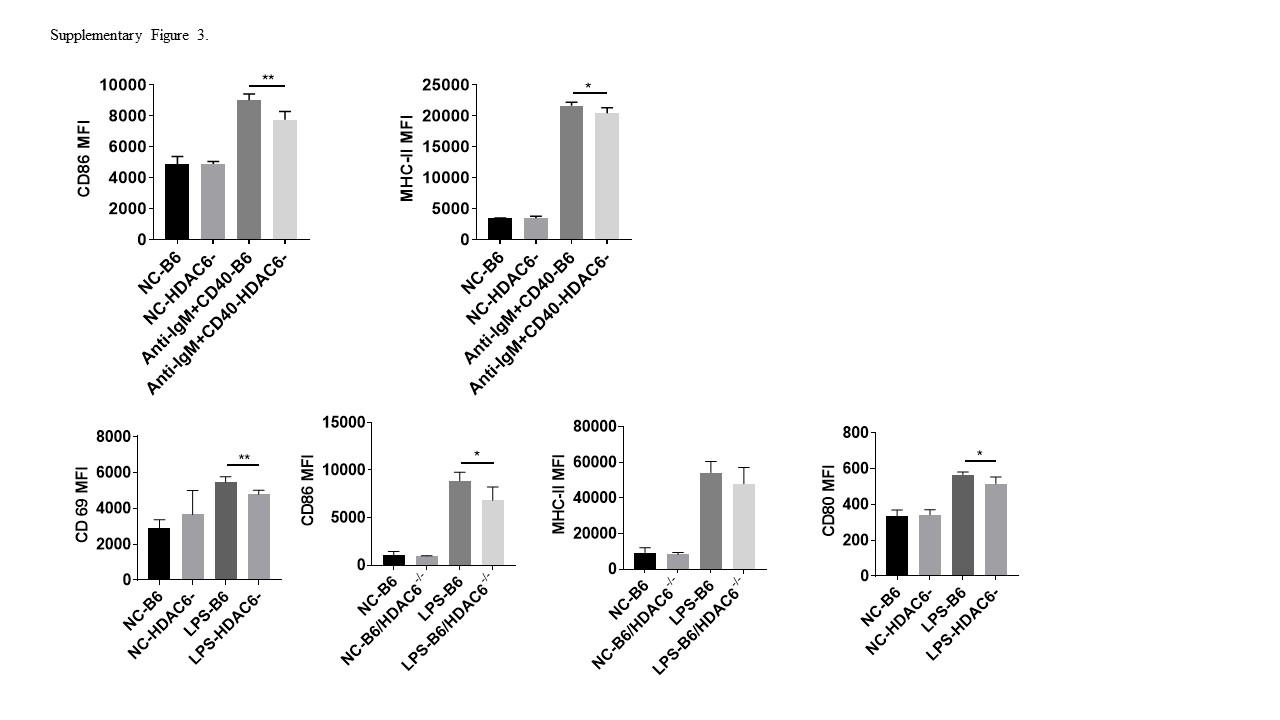

Supplement: Supplementary Figure 3 — Flow cytometry of sorted B cells from C57BL/6J mice and C57BL/6J/HDAC6−/− mice stimulated with LPS or anti-IgM, anti-CD40 for 24 h. The results showed reduced expression of activation markers of B cells CD86 and MHCII in C57BL/6J/HDAC6−/− mice compared to C57BL/6J mice with stimulation of anti-IgM and anti-CD40. In addition, MFI of CD69, CD86, and CD80 are downregulated in C57BL/6J/HDAC6−/− mice with stimulation of LPS. N = 5. *P < 0.05, **P < 0.01. [file Image_3.jpg]

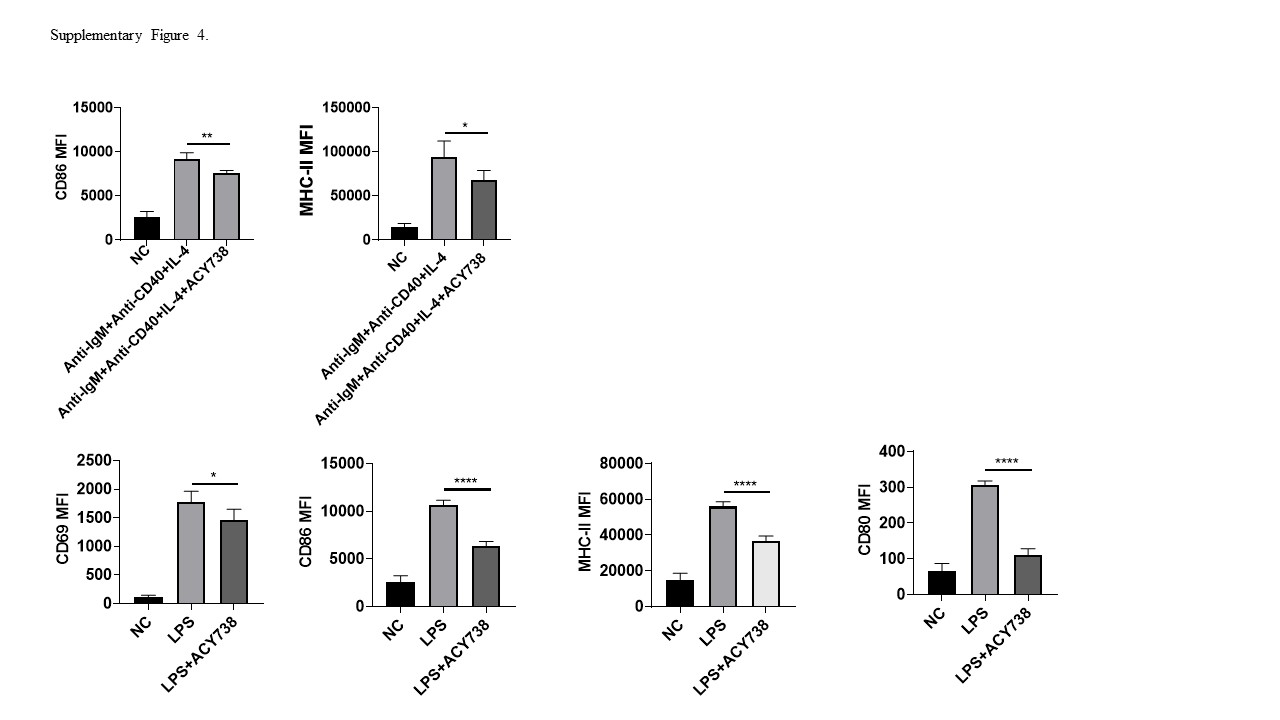

Supplement: Supplementary Figure 4 — Flow cytometry of sorted B cells from NZB/W mice stimulated with LPS or anti-IgM, anti-CD40 and then treated with ACY738 for 24 h. The results showed reduced expression of activation markers of B cells CD86 and MHCII in ACY-738 treated B cells with stimulation of anti-IgM and anti-CD40. In addition, MFI of CD69, CD86, MHC-II, and CD80 are significantly downregulated in ACY-738 treated B cells with stimulation of LPS. N = 5. *P < 0.05, **P < 0.01, ***P < 0.001, ****P < 0.0001. [file Image_4.jpg]

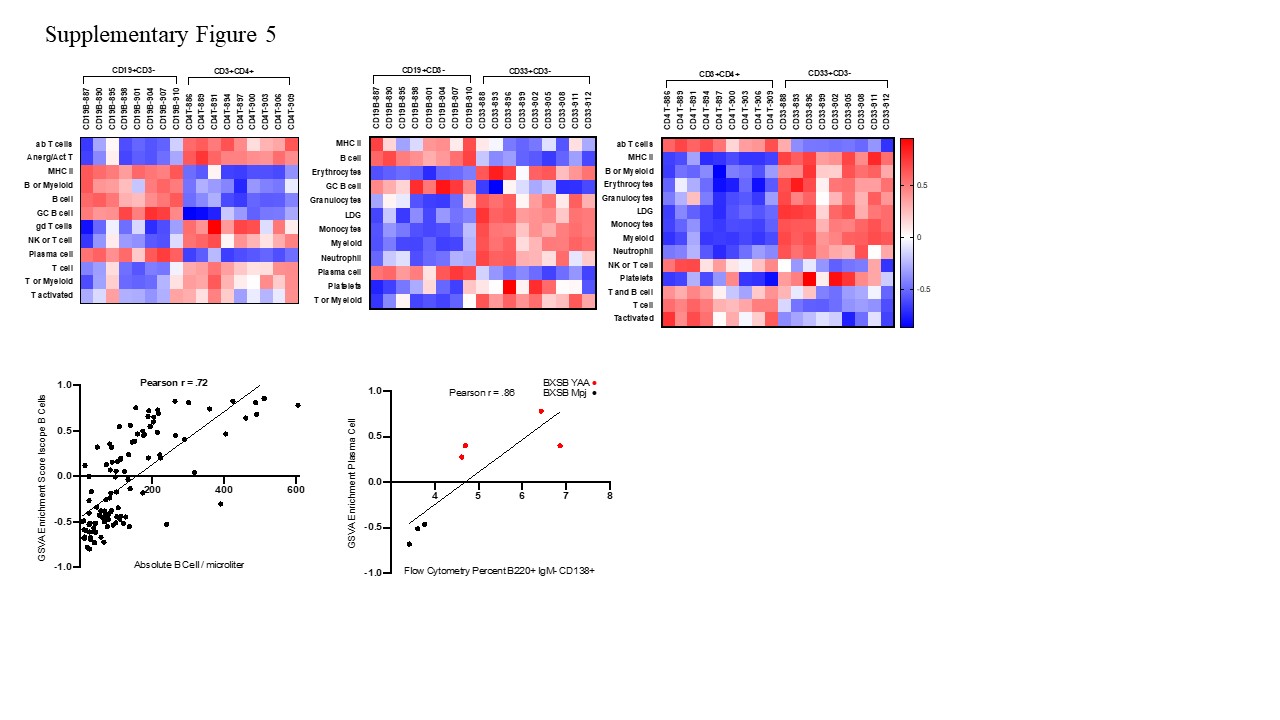

Supplement: Supplementary Figure 5 — (A) Control experiments demonstrate the specificity and lack of cross reactivity of I-scope. Experiments were performed on the DE analysis of healthy control purified CD3+CD4+ T cells, CD19+CD3− B and Plasma Cells, and CD33+CD3− Myeloid cells from microarray dataset GSE10325. The genes in each I-scope category (29 categories in total; hematopoietic general was not used) were used as modules for gene set variation analysis to determine the specificity of each module and cross-reactivity to other cell types. For each comparison, only categories with at least three genes above the Interquartile Range threshold were considered for statistical analysis. Significance of GSVA enrichment scores was determined using Sidak's multiple comparisons test. Adjusted p-values below 0.05 were considered significant. (B) Demonstration of strong relationship of human B cell/microliter counts to GSVA enrichment scores for the I-scope B cell category on 105 human subjects from microarray dataset GSE88884. Demonstration of the strong relationship of mouse flow cytometry values for plasma cells (B220+IgM−CD138+) and the GSVA enrichment scores using the I-scope plasma cell module on BXSB Yaa and BXSB MPJ mice. [file Image_5.jpg]
